# Supplementary material for: Are carbon emissions trading and green financial instruments synergistic? -Comprehensive quantitative research based on content analysis
Source: PLoS One. 2024 Mar 7;19(3):e0298601. doi: 10.1371/journal.pone.0298601 (PMC10919601; doi:10.1371/journal.pone.0298601)
Supplement: S1 Data — (ZIP) [file pone.0298601.s001.zip › Data/地级市Z_F工作报告.zip!/║╙─╧/║╫▒┌╩╨/└┤╫╘╣1⁄2╓┌║┼í╛┬φ┐╦╩2╛▌═°í┐_2011.docx]

各位代表：

　　现在，我代表市人民政府，向大会作政府工作报告，请予审议，并请各位政协委员提出意见。

一、2010年及“十一五”工作回顾

　　2010年是“十一五”规划的最后一年，也是我市经济社会发展不平凡的一年。在省委、省政府和市委的正确领导下，全市上下紧紧围绕率先崛起的奋斗目标，加快构建新型产业基础和新型城市骨架，坚持统筹产业、城乡、经济与生态、经济与社会协调发展，着力打好项目建设招商引资产业集聚区建设、新区建设和提升、老区改造和管理、新型农民社区建设、生态建设、职业教育六大攻坚战，胜利完成了市九届人大二次会议确定的目标任务，经济社会发展保持了好趋势、好态势、好气势。

　　经济在复杂形势下实现较快发展。初步核算，全市生产总值完成427.7亿元，比上年增长13.2%。工业经济运行良好，规模以上工业增加值260.5亿元、增长17.4%，实现主营业务收入913.4亿元、利润56.1亿元，分别增长34.2%和38%。固定资产投资快中趋优，全社会固定资产投资356.3亿元、增长24.2%，其中城镇投资305.3亿元、增长25%。财政收支较快增长，财政总收入34.2亿元、增长20.2%；一般预算收入22.2亿元、增长23%，一般预算支出59.1亿元、增长27.6%，财政支持发展的能力大幅提高。

　　新型产业基础构建取得重大成效。三位一体强力推进项目建设、招商引资和产业集聚区建设，促进产业扩规模、调结构。项目建设规模大、进度快、效果实。投资超5000万元的重点项目280个，当年完成投资260亿元、增长73%，其中超5亿元项目数量是上年的2倍；工业项目投资总规模占总量的80%左右；重点项目投资新增税收占税收增加额的近70%。招商引资实现历史性突破。实际利用境外直接投资突破两亿美元，引进省外资金超过90亿元，到位市外资金150.6亿元、增长51.4%，被评为全省对外开放工作优秀市、招商引资工作先进市、对外贸易工作先进市。中国石化、河南煤化投资170亿元的煤化一体化项目前期工作已全面展开，河南煤化鹤煤集团投资17.2亿元的1,4-丁二醇项目开工建设，与中国五环工程有限公司合作的煤制乙二醇中试项目正在投料试车，煤化工产业发展取得新突破。华晨集团一期投资12亿元的年产10万辆载货车项目全面开工，年产2万辆生产线已投产，一批汽车配套项目签约落地，汽车及零部件产业发展进入新阶段。中科院半导体研究所与深圳仕佳通信公司合作投资6.5亿元、填补国内技术空白的光分路器芯片项目开工，中国航空集团深圳航盛电子公司投资8亿元的汽车仪表及车载娱乐系统等40多个电子信息项目签约或开工建设，电子信息产业发展呈现良好势头。我市第一个国家级产品质检中心——国家镁及镁合金产品质量监督检验中心获批筹建；省镁合金特色产业园区加快推进，青岛地恩地投资10.2亿元、年产5万吨金属镁精深加工等项目加快建设，金属镁精深加工产业取得新进展。在新中源集团带动下，石林陶瓷园区总投资超50亿元的56条生产线建成9条、在建5条，一批配套企业落地建设，陶瓷产业初具规模，带动总投资45亿元的石林新城开工建设。产业集聚区综合带动作用更加凸显。4个省级产业集聚区完成投资138.4亿元，占城镇固定资产投资的45.3%；规模以上工业主营业务收入218.7亿元，从业人员3.75万人，分别占全市规模以上工业的23.9%和2.8%。开发区升格为国家级经济技术开发区，鹤淇产业集聚区被确定为全省首批新型工业化产业示范基地、创新型产业集聚区。质量效益进一步提升。二、三产业增加值占生产总值的88.6%，高技术产业增加值增长28.5%，规模以上工业企业增加68家，缴纳地方税收超100万元企业增加45家。金融机构新增贷款43.2亿元。市商业银行更名为鹤壁银行，具备了跨区域发展能力；村镇银行建设实现零的突破。天伦燃气公司在香港上市。

　　新型城市骨架构建迈出坚实步伐。大力推进城乡一体化，城镇化率达到51.8%，提高2.2个百分点。新区建设明显加快。在提升原有建成区品位的同时，加快框架拓展和功能完善，建成区达到29平方公里。“9+1”标志性建筑全部开工建设，其中5个主体封顶，产业综合服务功能区轮廓初步显现；淇水大道等主要道路建成通车，一批公共服务设施建成投用，南部片区框架已经形成。老城区改造全面推进。强力推进惠及3.3万户的地方城市棚户区改造，累计完成投资40多亿元、拆迁棚户区150万平方米，建设棚改小区35个、总面积240万平方米，已有1万多户喜迁新居，老城区集中供暖实现突破，棚户区改造工作得到国家、省充分肯定，得到全市尤其是老城区人民的大力支持。坚持政府引导、市场运作，吸引了江苏王房等战略合作者，计划投资100亿元参与老城区改造建设，老城区面貌正在明显改善。两县县城、重点镇和新型农民社区建设取得新进展。两县县城老城区改造提升和新城区建设加快推进，城市功能更加完善，承载能力明显增强。小城镇基本市政公用设施和公共服务设施启动建设，新型农民社区建成11个、在建22个。投资9.3亿元，改建新建高速、干线、农村公路301公里，鹤淇大道二期主体完工，快速通道北延一期（宝山大道）等工程竣工通车，群众出行更加便捷，城乡联系更加紧密。

　　现代农业优势进一步显现。粮食总产111.6万吨，连续6年创历史新高。3万亩粮食高产示范区小麦玉米亩产首创全国同面积高产纪录，5万亩示范区创建同步启动，为全国粮食生产探索了经验、提供了模式，全国高产创建现场会在我市召开。“玉米单交种浚单20玉米新品种选育及配套技术研究”获省科技进步一等奖。积极稳妥推进以专业合作社为主体的土地规模经营，对尚未就业且愿意流转土地的农民免费进行培训，确保每户家庭至少有1人就业；探索建立利益联结和风险防范机制，切实保障农民利益。温家宝总理在我市视察时，高度评价我市的农业生产特别是粮食高产创建、玉米育种、气象防灾减灾等工作，并将浚单系列玉米种子命名为“永优”，现代农业成为我市在全省乃至全国的一张名片。

　　生态文明建设全面扎实推进。覆盖全市的生态体系加快建设，淇河生态带建设顺利推进，森林公园、植物园、生态园等一批生态项目开工，全市完成造林20.1万亩，森林覆盖率24.8%。节能降耗目标任务圆满完成，循环经济标准化体系和标准评价体系进一步完善，6项循环经济标准成为国家标准。万元生产总值能耗下降5.6%。生态环境不断改善，化学需氧量排放提前完成“十一五”控制目标，二氧化硫排放量下降4.5%，空气质量优良率达到90%，河流出境断面、城市饮用水源地水质达标率均保持100%。

　　人民生活持续改善。全市财政投入39亿元改善民生，其中投入16.5亿元按期完成省、市民生工程。城镇居民人均可支配收入和农民人均纯收入分别达到15059元和6813元，增长10.5%和14.7%。就业工作扎实推进。城镇新增就业4.9万人，失业人员再就业2.2万人，动态消除了“零就业家庭”，更多群众就地就近就业。对所有初高中毕业后未升学人员实施了免费职业教育和技能培训。社会保障更加有力。企业退休人员养老金标准提高16.6%；新农合参合率99.4%，城镇居民基本医疗保险参保率84%。保障性住房开工建设13060套、竣工8643套，发放廉租住房补贴2386万元。解决了5.3万农村居民饮水安全问题。22个贫困村整村推进扶贫开发任务全部完成，7900人脱贫。社会事业全面发展。科技创新计划顺利实施，专利授权量增长47%。全省首家光电子技术院士工作站在我市挂牌成立。福田中学、鹿鸣中学、牟山小学建成投用，校舍安全改造5.7万平方米；实施素质教育，促进教育公平，“两基”水平稳步提高。鹤壁汽车工程职业学院大专申报顺利通过省高等院校评审委员会评审，职教园区被评为省级示范性职教园区。医药卫生体制改革稳步推进，全市所有政府办基层医疗机构实施了国家基本药物制度，基本药物价格下降20%～30%；在全省率先完成城乡居民健康档案建设工作、率先实现新农合门诊统筹，基层卫生服务体系进一步完善。市体育馆建成投用，市艺术中心主体完工。人口自然增长率控制在6.5‰以内。

　　社会大局和谐稳定。安全生产形势平稳，煤矿兼并重组在全省率先完成，被评为全省安全生产优秀市。强化产品质量和食品安全监管，无重大食品安全事故发生。积极排查化解矛盾纠纷，信访形势保持稳定，被评为全省信访工作先进市。平安建设深入推进，社会治安综合防控体系不断完善，现行命案发一破一，是全省2004年以来现行命案侦破率保持100%的省辖市，人民群众安全感、执法满意度综合排名均居全省第1位，荣获全省平安建设最高荣誉“中原平安杯”。

　　民主法制和精神文明建设不断加强。自觉接受人大及其常委会监督，接受政协民主监督，支持政协参政议政，认真办理人大代表建议和政协提案，办结率100%。法制建设全面推进，“五五”普法顺利完成。市县政府机构改革基本完成。强化行政监察、财政、审计等监督，深入推进反腐倡廉建设。加强精神文明建设，公众道德水平和文明程度不断提升。坚持“三具两基一抓手”，深入推进“两转两提”，认真开展“创先争优”活动，干部群众干事创业、团结拼搏的氛围更加浓厚。

　　大力开展国防教育，扎实做好双拥共建工作，军政军民团结进一步巩固。驻鹤解放军和武警部队在支持地方经济建设、维护社会稳定等方面作出了重要贡献。旅游、人防、外事、侨务、对台、民族宗教、统计、史志、档案、气象、地震等工作取得新成绩。四川江油市河口镇、东安乡灾后援建任务圆满完成。

　　各位代表！2010年经济社会的又好又快发展，确保了我市“十一五”主要任务胜利完成。“十一五”这五年是经济社会发展总体持续、综合实力大幅提升的五年，国民经济保持两位数增长，地区生产总值由不足200亿元跃上400亿元新台阶，人均生产总值由1600多美元到突破4000美元；财政总收入由不足15亿元到接近35亿元，一般预算支出由18亿元到接近60亿元。这五年是经济结构日趋优化、发展方式加快转变的五年，二、三产业增加值占生产总值比重比“十五”末提高6.1个百分点；城镇化率提高近10个百分点，成为全省第二个城镇化率超过50%的省辖市；万元生产总值能耗累计下降22%，化学需氧量和二氧化硫排放量分别累计下降11.2%和22%。这五年是粮食产量连创新高、高产创建不断突破的五年，粮食产量由“十五”期间年均85.1万吨增加到“十一五”连续五年超百万吨；高产创建万亩核心示范区在全国率先实现万亩连片小麦、玉米一年两熟亩产超吨半粮目标，创建面积逐年扩大，科技含量不断提高。这五年是基础设施加快建设、支撑能力明显增强的五年，累计完成全社会固定资产投资由203.6亿元增加到1120.5亿元，盘石头水库下闸蓄水，发电装机总规模由115万千瓦增加到220万千瓦，公路总里程由1611公里增加到4341公里。这五年是城乡面貌显著改变、生态环境持续优化的五年，市区建成区面积由40.4平方公里增加到50.9平方公里，新区规划面积由38平方公里增加到65平方公里，150平方公里左右的“大新区”正在规划，两县县城城区面积增加26.1%；以淇河生态带为重点的生态体系建设进展较快，林木覆盖率由21.2%提高到27.2%。这五年是社会事业全面进步、人民群众受益更多的五年，每年实施一批民生工程，累计投入111.2亿元用于改善民生，占当期财政一般预算支出的60%，比“十五”时期增加87.2亿元；老城区改造全面启动，采煤沉陷区综合治理和鹤煤棚户区改造工程基本完成、近8万群众受益，在全省率先启动地方城市棚户区改造工程、惠及15万人；累计新增城镇就业24万人；义务教育投入22.1亿元，年均增长31%；公共医疗卫生支出12.3亿元，年均增长38.6%；3.6万贫困人口脱贫；解决农村29.7万人的饮水安全问题；公共服务体系和社会保障体系不断健全，并加快向农村延伸；城镇居民人均可支配收入由7899元增加到15059元，农民人均纯收入由3469元增加到6813元，均接近翻一番。

　　各位代表！五年来，我们一步一个脚印扎实工作，积极应对各种复杂局面，有效化解国际金融危机等不利影响，经济社会实现了跨越式发展，全市人民有理由为此感到自豪！这是省委、省政府和市委正确领导的结果，是市人大、市政协鼎力支持的结果，是全市人民团结拼搏的结果。在此，我代表市人民政府，向工作在全市各条战线上的工人、农民、知识分子、干部、驻鹤解放军指战员、武警官兵、公安民警以及各界人士致以崇高的敬意！向所有关心、支持我市现代化建设的朋友们表示衷心的感谢！

　　五年的成绩来之不易，积累的经验弥足珍贵。通过实践，我们进一步深化了对科学发展观的认识，增强了贯彻落实科学发展观的能力。我们深刻体会到：必须把发展和为民作为治市理政的最高价值标准，把发展作为解决前进中一切问题的关键，努力在发展中调整、转变、提升、增效；突出为民，把发展经济与改善民生紧密结合，把扩大投资与加强民生工程建设统筹推进，让改革发展成果最大程度惠及广大人民群众。必须把鹤壁发展放在全省、全国大局中去谋划运作，着眼全局找准定位、积极融入，坚持在服务大局中谋求发展，在为全省和中原经济区建设作贡献中实现鹤壁更好更快发展。必须把重在持续作为关系长远的战略方针，坚持不懈地把两个构建推向前进，并着眼长远打基础、增后劲，通过发挥传统优势、培育新兴优势，创造我市在区域竞争中的比较优势，增强综合竞争力。必须把开放带动作为主战略、项目建设作为主抓手，今后一个较长时期，鹤壁经济社会发展的主动力依然是投资，而投资必须坚持招商这个主渠道，牢牢抓住项目建设这个主抓手，把智慧、资源和力量集中到项目谋划、引进和建设上。必须把社会治安、信访、安全生产形势基本平稳作为重要保障，我市“十一五”取得的成绩，与社会大局和谐稳定密不可分，必须高度自觉、积极主动地抓好平安建设，为经济社会发展创造良好环境。这些经验值得我们在今后工作中坚持和发扬。

　　各位代表！在肯定成绩的同时，我们也清醒地认识到，我市产业单、链条短、规模小的深层次结构问题尚未根本解决；老城区改造发展任务艰巨；经济发展与环境保护的矛盾仍然突出；管理人才和专业技术人才短缺；政府工作还存在不少问题和不足，部分干部思想有待进一步解放、素质有待进一步提高、作风有待进一步改进。对这些问题，我们将以对党和人民高度负责的态度，求真务实、开拓创新，通过艰苦细致的工作和坚持不懈的努力加快解决。

二、“十二五”发展总体思路和目标任务

　　市委七届八次全会通过的创建科学发展示范区纲要和“十二五”规划建议，是指导鹤壁今后一个时期发展的行动纲领。创建科学发展示范区的总体目标是：在探索工业化城镇化农业现代化协调发展和人与自然、人与社会相和谐的科学发展路子中走在前面、作出示范。具体目标是：产业结构优化，产城布局合理，综合效益较高，城市绿色低碳，社会稳定和谐，体制机制健全。具体分两个阶段推进：第一阶段也就是“十二五”时期，力争通过5年左右的努力，构建起新型产业基础和新型城市骨架；第二阶段是再经过5年乃至更长时间的努力，构建起新型产业体系和新型城镇体系，在全省率先实现全面建设小康社会目标，基本建成科学发展示范区。

　　“十二五”时期是我市可以大有作为的重要战略机遇期，是加快两个构建、创建科学发展示范区、加快率先崛起步伐的关键期，是负重爬坡、不进则退、务求突破的攻坚期。这一时期，我们面临国家扩大内需、我省建设中原经济区、沿海产业向内地加快转移、我市经济社会发展保持良好势头等难得历史机遇，尤其是今年温家宝总理莅临我市视察，充分肯定了我市工作，指明了我市发展方向，给我们信心、给我们力量，全市人民深受鼓舞、倍感振奋；同时我们也面临着结构性矛盾突出、资源环境约束加剧、区域竞争压力加大、改善民生任务繁重等诸多困难。总体上看，有利因素多于不利因素。我们要抢抓机遇、乘势而上，集中力量、克难攻坚，确保“十二五”开好局、起好步。

　　“十二五”时期我市经济社会发展的指导思想是：高举中国特色社会主义伟大旗帜，以邓小平理论和“三个代表”重要思想为指导，深入贯彻落实科学发展观，按照建设中原经济区、加快中原崛起和河南振兴战略部署，以创建科学发展示范区、加快率先崛起步伐为总战略，以加快构建新型产业基础和新型城市骨架为总任务，以富民强市为根本，以统筹产业、城乡、经济与生态、经济与社会协调发展为总原则，坚持既定思路目标举措不变调、不争论、不折腾、不懈怠，为全面建成小康社会打下具有决定意义的基础。

　　今后五年我市发展的蓝图和愿景是：经济实力显著提升，在优化结构、提高效益和降低能耗的基础上，确保经济持续平稳较快发展，主要经济指标年均增速保持全省先进水平，主要人均指标在全省位次前移；全市生产总值年均增长12%左右。经济结构显著优化，城乡结构、产业结构、要素投入结构调整取得重大进展，“三化”发展的协调性不断增强，经济发展的质量和效益明显改善；财政总收入力争实现翻一番；全社会固定资产投资累计实现翻一番；二、三产业增加值占比达到90%以上；城镇化率达到60%以上。居民收入显著增加，努力实现居民收入增长和经济社会发展同步，劳动报酬增长和劳动生产率提高相协调，农民人均纯收入继续保持全省先进水平，城镇居民人均可支配收入力争达到或超过全省平均水平，人民生活质量和水平较大提升。生态环境显著改善，努力实现生态改善与产业发展同步，资源节约、环境友好型社会建设迈出较大步伐，以淇河生态带为重点的生态体系基本形成，循环经济发展取得重大进展；万元生产总值能耗累计下降16%，主要污染物排放量控制在省下达目标内，森林覆盖率达到28%，工业固体废物综合利用率达到95%。社会建设显著加强，努力实现社会进步与经济发展同步，就业岗位持续增加，覆盖城乡居民的社会保障体系逐步完善，基本公共服务均等化程度提高，公民思想道德素质、科学文化素质和健康素质不断提高；文化事业和文化产业加快发展；民主法制更加健全，人民权益得到切实保障，社会管理制度逐步完善，社会更加和谐稳定。改革开放显著突破，政府职能转变、财税金融、要素价格等重要领域和关键环节改革取得明显进展，政府公信力和行政效能进一步提高，开放广度深度不断拓展。

　　《鹤壁市国民经济和社会发展第十二个五年规划纲要（草案）》及说明已印发各位代表，请予审议。

　　我们坚信，有省委、省政府和市委的正确领导，有全市广大干部群众的团结拼搏，我市“十二五”发展蓝图一定能够如期实现！

三、2011年发展目标和重点工作

　　今年是“十二五”的开局之年、创建科学发展示范区的起步之年。我们要认真贯彻落实温家宝总理视察我市时的重要讲话精神和市委七届八次全会、经济工作会议精神，以科学发展为主题，以加快转变经济发展方式为主线，围绕创建科学发展示范区、加快率先崛起步伐的总战略，加快两个构建，坚持四个统筹，突出项目建设和开放带动，全面提高发展的质量和效益，以全新的精神状态，努力创造新优势，奋力实现新突破，促进经济社会更好更快发展。

　　主要预期目标是：生产总值增长11%；规模以上工业增加值增长15%；全社会固定资产投资增长21%，其中固定资产投资增长23%；财政一般预算收入增长12%；社会消费品零售总额增长16%；引进市外资金160亿元；人口自然增长率控制在6.5％。以内；城镇新增就业3万人以上；城镇化率提高1.8个百分点；城镇居民人均可支配收入增长9%，农民人均纯收入增长9%；万元生产总值能耗、主要污染物排放量完成省下达控制目标。

　　实现这些目标，是政府各级各部门的责任和使命，更是人民群众的热切期待，我们一定圆满完成这些目标，并努力向更高目标迈进！

　　今年重点抓好以下几个方面的工作：

　　（一）全力实现两个构建新突破，推进“三化”协调发展

　　今年是实现两个构建的关键之年，各项工作都必须科学谋划、克难攻坚，保持好势头、实现新突破。

　　一是务求新型产业基础构建实现新突破。加快培育壮大主导产业和战略性新兴产业，力争规模以上工业增加值超过300亿元，企业主营业务收入突破千亿元大关。做大做强主导产业。煤化一体化方面，坚持搭好平台、密切配合、全力服务，争取煤化一体化项目尽早开工、顺利推进；年产60万吨甲醇项目建成投产，1,4－丁二醇项目土建工程基本完成，氯碱循环经济项目试生产，力争煤制乙二醇一期工程、聚甲醛项目开工建设，大力开发下游产品，推动产业链向高端延伸。汽车及零部件方面，华晨载货车项目年内形成10万辆产能，同时加快推进汽车零部件配套园区建设。食品工业方面，下大力气推进一批重点项目，引进2～3家龙头企业，在扩大规模、提升层次、打造品牌上实现新突破。培育壮大战略性新兴产业。金属镁精深加工方面，积极推进国家镁及镁合金产品质量监督检验中心建设，大力发展高品质镁合金挤压型材、汽车和手机镁合金部件等高附加值产品，加快建设省镁合金特色产业园区，全年金属镁综合加工规模达到15万吨。电子信息方面，积极推进光电子产业园建设，争取光分路器芯片项目试生产，航盛公司汽车仪表及车载娱乐系统项目部分投产。继续引进一批陶瓷和配套企业，年内再投产12条、开工建设10条生产线，加快建设规模大、技术含量高、影响力强的新型建筑陶瓷生产基地；积极发展纺织服装、节能环保、新能源等产业，尽快形成生产能力和核心竞争力。大力发展现代农业。认真落实强农惠农政策，努力用现代物质条件装备农业，用现代科学技术改造农业，用现代经营形式推进农业，建设现代农业示范市。坚持稳定总产、主攻单产、改善品质、提高效益，以2个5万亩、1个3万亩、5个1万亩高产创建示范方为带动，积极推进整乡整县建制高产创建，丰富高产创建内涵，提高粮食综合生产能力；谋划推进水利等农业基础设施建设，积极推进火龙岗23万亩节水灌溉等工程，实现节水灌溉与高产创建同步推进。加快农业结构调整，大力发展现代畜牧业，进一步加大畜产品质量监管和重大动物疫病防控力度，确保畜产品质量和畜牧养殖业安全；积极发展高效农业、特色农业、生态农业，推进一批特色产业基地建设，多策并举抓好“菜篮子”工程；以“永优”浚单系列为重点，加大玉米新品种研发推广力度，打造鹤壁玉米种业品牌。完善星陆双基农田信息协同反演技术和人工影响天气指挥系统，提高农业信息化、数字化水平，增强农业稳定增产和防灾减灾能力。大力培育农业龙头企业，规范、支持农民专业合作组织和农业社会化服务体系建设，积极探索推广农业产业化运作模式，稳妥推进农村土地流转，提高农业产业化水平，增加农民收入。积极发展服务业。扎实推进服务业综合改革试点工作，面向更广区域，丰富提升传统服务业，突出发展新型商贸、现代物流、金融、旅游等现代服务业，积极引进新型商业业态，大力推进服务业项目建设和高成长性服务业企业发展，58个服务业项目完成投资54亿元。加快建设冷链、煤炭、陶瓷等物流项目和国际陆港，培育第三方物流。推进旅游景区基础设施建设，完善配套服务功能，提高我市旅游景区的吸引力。

　　二是务求新型城市骨架构建实现新突破。加快推进城镇化，统筹新老城区建设、城乡建设协调发展，大力实施城乡建设2011年行动计划，突出工作重点，创新理念机制，强化服务管理，全面提升规划、建设、管理水平。加快建设“大新区”。完成“大新区”建设方案的编制和报批，加快推进鹤淇一体化，充分发挥核心和辐射、带动作用。强力推进新区南部片区建设，产业综合服务功能区完成投资9亿元，5座标志性建筑竣工，市艺术中心、高铁车站等项目竣工投用，淇河防渗减漏工程完工，启动重点产业服务区、专业市场园区基础设施建设，积极推进钜桥商贸区建设。加快推进新区建成区11个城中村改造，4个城中村居民搬入新居。启动市行政服务中心搬迁，建设数字鹤壁综合服务中心，拓展服务功能、提高服务层次。新建续建道路21.7公里，加大二支渠、棉丰渠、天赉渠治理改造力度，完成护城河治理，基本完成15个主要积水点改造。继续提升新区城市品位，加快农贸市场、汽车站点、公厕等公共服务设施改造建设，全面提高社区物业等城市服务管理水平；科学规划、优先发展城市公交，打造“15分钟生活圈”，方便群众生活。加快老城区和两县县城改造发展。以棚户区改造带动和促进保障性住房建设、沿街危旧房改造、城中村改造，全面提升改造建设管理水平，力争改造一个小区就建成一个精品，进一步改善老城区群众居住条件。启动6个城中村、2个社区改造；加大供水、供电、供气、供暖、绿化等基础设施以及集贸市场、文化体育设施等直接关系民生的公共服务设施建设力度，努力使今年成为老城区改造建设大推进、大提升的一年，使老城区群众享受到更多更优的公共服务。加快两县老城区改造、新城区建设，基础设施和公共服务设施完成投资42.5亿元，明显改善县城面貌，进一步提升综合承载能力。协调推进小城镇和新型农民社区建设。以善堂、王庄、石林为重点，大力推进小城镇基础设施和公共服务设施建设。积极稳妥推进35个新型农民社区建设。同时，坚持城乡统筹、全域谋划、产城互动，高效率、高质量编制城乡一体化、老城区统筹建设发展、鹤淇一体化空间发展、城市综合交通体系、新型农民社区等规划设计，加强城市风格定位研究、规划专项研究和重点项目规划服务；强化城乡规划全过程联合监管和施工管理，严肃查处违法违规建设，保证城乡建设的质量和品位。

　　三是为两个构建实现新突破提供有力支撑。坚持以项目建设为主抓手。全领域推进项目建设，着力抓好360个5000万元以上重点项目，完成投资310亿元以上，竣工投产85个，使今年成为项目建设扩总量、提质量、增效益的一年。继续做好南水北调中线工程、石武客运专线、山西中南部铁路通道等过境项目的协调服务工作，确保项目在鹤壁境内顺利推进。加快推进鹤辉高速公路、葛嘴线中段改建、快速通道北延二期等工程，力争107国道鹤壁段改造开工，积极做好鹤壁至水冶高速公路、渤海路向金山园区西延等前期工作。科学谋划一批符合国家政策导向、市场前景好、带动能力强、投资超20亿元的重大项目。坚持以开放招商为主渠道。牢固树立开放决定成败的观念，高度自觉地抓好以承接沿海产业转移为中心的招商工作，进一步强化领导力量，保障时间精力，优化体制机制，提升运作水平和招商实效。围绕主导产业和战略性新兴产业，突出重点产业、重点企业、重点区域，继续开展定向定位定点坐地招商，吸引企业链条式、板块式转移，促进产业集聚并向高端发展，提升市场竞争力。重点抓好已签约项目的跟踪推进，加强与中国石化、河南煤化等大企业、大集团的合作，把资源集中运用到行业龙头和产业集群上，力争今年有2～3家行业龙头落户我市。认真研究用好国家、省政策，积极争取上级资金。坚持以产业集聚区为主载体。快速推进道路、供排水、污水垃圾处理、标准厂房、职工宿舍等基础设施和公共服务设施建设，确保所有集聚区具备“五通一平”条件，已经具备的加快向更高标准推进。创新完善推进机制和服务管理体系，统筹全市项目集中向产业集聚区和产业园区摆放、异地项目向专业园区摆放。基础设施项目完成投资40亿元，工业项目完成投资110亿元，主营业务收入达到400亿元，保持项目建设、产业发展在全省的特色和优势。开发区以升格为国家级经济技术开发区为契机，持续增强开放创新活力和发展内生动力，实现二次创业、跨越发展，真正成为对外开放的窗口，成为经济社会发展的增长极、示范区、排头兵。坚持以现有企业为主平台。统筹推进现有企业发展壮大和招商引资、项目建设，促进企业扩规模、上水平、创品牌。积极扶持优势企业，争取进入省“双百”行列。加强规划研究、分类指导，促进中小企业加快集聚，不断发展壮大。市、县两级继续服务好100家重点企业，落实和完善政策导向、融资服务、市场开拓、困难企业帮扶等服务企业长效机制，帮助协调煤电油气运等生产要素，深入推进产销对接，鼓励企业家进一步开阔眼界、勇于创新，促进企业做大做强做优。深化政府、银行、企业合作，积极引进域外金融机构，加快培育农村新型金融机构，继续大力发展金融市场，优化金融生态环境，打造发展的金融、开放的金融、创新的金融、诚信的金融。担保机构为中小企业融资6亿元以上。积极争取1家企业上市。力争发行城市建设投资债券。深入实施质量立市战略，支持企业技术改造和新产品研发，努力培育一批骨干企业和知名品牌。大力实施标准化战略，支持优势产业、重点企业将具有自主知识产权的技术和专利及时转化为标准，将具有比较优势的企业标准上升为国家标准。坚持以改革创新为主动力。深化行政管理体制改革，创新公共服务供给机制，提高服务水平。稳步推进教育教学、医药卫生体制、事业单位人事制度和养老保险制度等改革。大力发展非公有制经济，鼓励和引导民间资本进入基础产业、基础设施、社会事业、金融服务等领域。加快水利体制机制改革，实行最严格的水资源管理制度，促进水资源优化配置和节约集约使用。继续实施科技创新计划，与省相关厅局建立厅市合作机制，与科研院所和高校加强合作，加快光电子技术院士工作站、博士后研发基地建设。实施重大科技专项，尽快形成一批支撑企业发展的关键技术。加快培育一批创新型企业和高新技术企业，新建工程技术研究中心10家、创新型试点企业15家，加快提升企业科研能力和核心竞争力。

　　（二）着力打造鹤壁特色品牌，再创率先崛起新优势

　　在发挥好已有比较优势、打造现代产业品牌的同时，努力打造我市特色品牌，增强对外吸引力和影响力。

　　一是打造开放创新品牌。坚持用开放的视野、思路和举措，谋划发展、推进工作，学习借鉴外地的好经验、好办法，善于整合方方面面的资源为我所用。坚持尊重规律、实事求是，敢于突破条条框框、行政区划的限制，善于向体制机制创新要办法、要实效。培育主体、完善载体、打造平台，加大物流、劳动力、配套、人才、公共服务等领域的改革创新力度，全面建设创新型鹤壁，加快发展方式从要素驱动型向创新驱动型转变。

　　二是打造人力资源品牌。把服务鹤壁与面向全省全国有机结合，继续大力发展职业教育，搞好劳动力培训。加快职教园区和鹤壁职业技术学院新校区建设，争取鹤壁汽车工程职业学院等5所职业院校实现招生，高等职业教育在校生达到1.5万人。对“两后”人员进行不少于3个月的技能培训并推荐就业；搞好农民工尤其是失地农民培训教育，适龄失地农民培训率达到90%，促进农民工加快向产业工人转变。继续实施人才引进计划，督促各县区和开发区落实和完善政策，更加开放地引进多层次创新创业人才，支持企业引进基层管理人才、高技能人才，培育和用好现有人才，创造人尽其才的良好局面。

　　三是打造服务环境品牌。坚持人民至上、企业至上、服务至上，对与人民群众生产生活、企业经营发展紧密相联的事，不说不能办、只说怎么办，完善联审联批、全程代办、专案研究等措施，切实做到高效服务、精细服务、诚信服务、阳光服务、创新服务。把更多时间和精力放在改革发展一线，有针对性地解决实际问题，全面提升服务经济社会发展的质量和水平，真正使服务成为我市第一竞争力。

　　四是打造生态宜居品牌。把生态宜居理念贯穿城市规划、建设、管理的各个环节，坚持以人为本，完善城市设施，加强服务管理，丰富文化内涵，吸引更多的人到鹤壁生活、发展。持续抓好林业生态示范市和国家森林城市建设，重点推进总投资21.6亿元的淇河生态绿化等10个生态项目，保护好淇河这条生态河、我们的母亲河；高质量完成造林18.2万亩，森林覆盖率达到26%。广泛深入开展公民道德建设和文明创建活动，提升公民文明素质和城乡文明程度。按照覆盖面广、技术标准高、功能应用全、服务支撑强的原则，全面启动“数字鹤壁”建设，加快构建全市统一的政务网络和数据中心平台，力争年内建成框架，初步实现信息技术资源共享，使群众享受更多数字化服务带来的便利和实惠。

　　五是打造循环经济品牌。强化绿色低碳理念，积极创建全国循环经济示范市和循环经济标准化示范市，争取成为国家资源型城市可持续发展试点。抓好循环经济项目建设，力争20个续建项目全部竣工、12个计划开工项目全部开工。积极发展煤矸石无机纤维为主导的无机新材料等产业，促进煤炭的综合利用和延链增值。高标准建设宝山循环经济产业集聚区。完善城市可再生资源回收利用体系。进一步提升循环经济标准化水平。大力开展工业节能，发展建筑低碳和节能产业，淘汰落后产能。加大既有建筑节能改造投入，积极推进新建建筑节能和低碳社区建设，完成可再生能源建筑应用示范项目150万平方米，通过新能源形式新增供热面积100万平方米。全面推广清洁生产，抓好污染物总量减排，大力实施农村环境综合治理，科学系统地调配使用水资源，严格保护饮用水源地，省控河流出境断面水质达标率80%以上，空气质量优良天数310天以上，进一步改善环境质量。

　　六是打造特色文化品牌。保护城市历史文脉，弘扬传统文化，塑造开放、创新、淳朴、博爱等鹤壁核心文化标识。以文化改革发展实验区为载体，加强城乡基层文化基础设施建设，结合现代文化消费需求重点谋划推进一批文化项目，发展特色文化事业和产业，培育新型文化业态。深入挖掘淇河文化、民俗文化、鬼谷子文化，科学策划、提升层次，促进文化旅游事业发展。积极配合做好大运河鹤壁段申报世界文化遗产工作。弘扬牛派艺术，创造思想深刻、艺术精湛的精品。继续实施文化惠民工程，增强公共文化产品供给和服务能力，更好地满足人民群众文化需求。

　　（三）切实保障和改善民生，不断提升公共服务水平

　　把提高居民收入、改善群众生活摆在更加突出的位置，更加注重城乡公共服务的一体化、均衡化，全年市级财政用于民生的投入增长18.5%，让人民群众享受更多改革发展成果。

　　一是扎实推进民生工程。建设各类保障性住房37084套，其中开工建设廉租住房18198套、公共租赁住房5200套、经济适用住房560套，城市和国有工矿棚户区改造住房13126套，发放廉租住房补贴8809户。在搞好试点的基础上，多方筹集资金解决好偏远山区道路、饮水安全等问题，有计划有步骤地改善深山区群众生产生活条件。落实调整收入分配关系的各项政策，着力提高城乡低收入群体收入，扩大中等收入群体。完成事业单位实施绩效工资工作。城市社区工作人员工资标准提高到每月800元以上，农村党支部书记、村委会主任、其他“两委”干部每月工作报酬均增加100元，分别提高到600元、500元、400元，对城市新建社区的扶持标准由每个10万元增加到15万元。物价问题涉及民生、关系全局、影响稳定，要认真落实监管措施，努力稳定市场物价。加快农村基础设施建设，解决农村1万人饮水安全问题，新建户用沼气4000户、大中型沼气工程3座，改造升级一批农村公路、主要干线公路和桥梁，进一步改善群众生产生活条件。

　　二是切实做好就业工作。继续实施更加积极的就业政策，大力发展劳动密集型产业、服务业、小型微型企业和创新型科技企业，扶持自主创业和自谋职业，努力满足不同层次的就业需求。加强技能培训，完善信息服务，实施零就业家庭专项援助和公益性岗位安置计划，重点解决高校毕业生、城镇就业困难人员、退役军人、农村转移劳动力的就业问题，在确保完成城镇新增就业目标的同时，下岗失业人员再就业1万人，失地农民就业率达到95%以上。加强劳动保障监察执法，依法维护劳动者权益，构建和谐劳动关系。

　　三是加快建设覆盖城乡居民的社会保障体系。继续扩大养老保险覆盖面，将未参保集体企业退休人员全部纳入基本养老保险。扩大城乡低保覆盖面。健全企业退休人员基本养老金、城乡居民低保、农村五保供养、优抚对象和孤儿基本生活最低养育标准正常调整机制。落实基本养老保险关系跨地区转移接续办法。完善城镇职工、居民医疗保险制度和新农合制度，继续扩大基本医疗保障覆盖面，新农合参合率保持95%以上，城镇居民参保率达到85%以上；新农合和城镇居民医保财政补助标准提高到200元，新农合最高补偿限额提高到10万元，人均基本公共卫生服务经费标准提高到25元；全面实现城镇基本医疗保险即时结算和医疗保险、工伤保险市级统筹，实现新农合跨区域即时结报；完善城乡居民健康档案。加大扶贫济困力度，扩大城乡困难群众受助范围。切实解决失地农民生活保障问题，实现失地农民社会保障制度全覆盖。发挥好商业保险在完善社会保障体系中的作用。加快建设完善社会福利、殡仪服务等设施。大力发展慈善事业。广泛开展志愿者活动。

　　四是全面发展社会事业。26个重点社会事业项目完成投资12亿元。完善义务教育经费保障机制；实施校舍安全改造5.4万平方米；整合建设中小学特别是农村学校，高标准建设农村寄宿制学校；巩固“两基”水平，推行素质教育，加强中小学教辅书的监督管理，保证中小学生每天1小时校园体育活动；坚持城乡一体、统筹布局和标准、公办民办并举，增加学前教育资源，基本普及学前一年教育，确保每个乡镇至少有1所办学条件较好的幼儿园。全面实施国家基本药物制度，建立完善基本药物保障供应体系，切实降低药价，加强药品监管，确保用药安全；完成农村三级卫生服务网络和城市社区卫生服务机构建设任务，继续推进县级医疗机构、中心乡镇卫生院和标准化村卫生室信息化建设；加强重大传染病、慢性病、职业病、地方病、精神疾病的预防控制和规范管理；整合医疗卫生资源，积极创建三级甲等医院，扶持特色专科医院发展，为人民群众提供更多更好的医疗服务。实施体育设施城乡全覆盖工程，广泛开展全民健身活动，提高健康素质。稳定低生育水平，统筹解决人口问题，促进人口全面均衡发展。切实保护妇女儿童权益。做好老龄化应对工作，积极发展康复养老、家政服务等产业。建设少数民族群众公墓。

　　健全突发事件应急体制，加强地震应急避难场所等公共安全应急设施建设，提高全社会危机管理和防灾减灾救灾能力。

　　五是努力保持社会和谐稳定。加强和改进群众工作，深入推进社会矛盾化解、社会管理创新、公正廉洁执法三项重点工作，健全、落实信访工作责任制，做好舆情分析和舆论引导，切实解决群众反映强烈的问题。加强法制宣传教育，启动“六五”普法规划。深入推进安全鹤壁建设，落实企业安全生产和产品质量主体责任，完善安全生产监管监察体制，紧紧抓住安全投入、管理和培训三项基础工作，坚决遏制重特大安全事故发生。强化责任，完善机制，严格执法，全面提高食品安全保障水平。扎实推进平安建设，完善社会治安防控体系，加强社会治安综合治理，加大人防、技防投入，严密防范、依法打击各类违法犯罪活动，保持社会治安、信访、安全生产形势基本平稳，让人民群众生活得更加安心、放心、舒心。

　　加强国防教育和国防后备力量建设，支持驻鹤解放军、武警部队、民兵预备役和人民防空建设，促进军民融合发展。深入开展双拥共建，巩固和发展军政军民团结。支持工会、共青团、妇联等人民团体发挥桥梁纽带作用。做好外事、侨务、对台、民族宗教、统计、史志、档案等工作。

　　四、全面加强政府自身建设

　　坚持“三具两基一抓手”，深入推进“两转两提”，扎实开展“创先争优”和“做鹤壁人民好公仆”主题教育活动，努力为人民办实事、办好事，建设人民满意的政府。

　　坚持执政为民。进一步提升为民认识，始终把人民群众的需要作为工作的第一信号，把人民群众的满意作为工作的唯一标准。突出为民发展，通过推动经济社会更好更快发展，促进各项民生事业不断进步。健全为民机制，完善民意反映、民主决策、群众权益维护等制度，准确把握群众所想、所忧、所盼，有效保障人民群众权益。树立为民形象，以人民利益为重，在工作谋划部署尤其是人力物力财力安排上，更多地向民生倾斜，更好地为人民群众服务。

　　坚持依法行政。切实按照法定权限和程序行使权力、履行职责，做到严格规范、公正文明执法。严格执行人大及其常委会的决议、决定，依法接受其法律和工作监督，主动接受政协民主监督，支持政协参政议政，认真办理人大代表议案、建议和政协提案，充分听取各民主党派、工商联、无党派人士及各人民团体的意见，接受社会监督。深化政务公开，积极推进财政预算等信息公开。坚持环境定期评价制度，健全行政效能投诉受理督查反馈机制，强化行政问责，坚决治庸治懒，严查行政慢作为、不作为和乱作为。

　　坚持转变职能。以领导方式转变促进发展方式转变，强化社会管理和创新，努力实现政府职能向创造良好发展环境、提供优质公共服务和维护社会公平正义转变。注重发挥政府资源和财政资金的引导作用，发挥市场和社会力量的主体作用，发挥信息网络等现代化手段的支撑作用，服务和促进经济社会发展。加强农村基层组织和城市社区建设，实现重心下移，夯实基层基础。把管理和服务更好地结合起来，在服务中实施管理，在管理中体现服务。

　　坚持求实求效。突出运作、狠抓落实，说到做到、说好做好，提高政府执行力和公信力。带着感情深入实践、深入一线，虚心向群众请教，多帮助群众、企业解决实际问题。注重统筹协调和资源整合，提高财政资金使用质量，为人民用好每一分钱，降低发展和行政成本，建设节约型政府。加强公务员队伍教育管理，大力推进学习型机关建设，进一步提高为人民群众服务的能力和水平。

　　坚持廉政勤政。加强廉政建设和反腐败工作，认真治理政府工作人员以权谋私和渎职侵权问题，坚决纠正损害群众利益的不正之风。坚决制止铺张浪费和形式主义，严格控制一般性支出，公务接待、因公出国（境）、公务用车购置及运行维护三项经费支出预算原则上实行零增长，精简会议、文件，减少不必要的应酬和检查评比。严格执行领导干部廉洁自律规定，严格教育、管理、监督干部，使广大干部切实做到警钟长鸣、秉公用权，坦坦荡荡做人、干干净净做事，把精力最大限度地用在为人民勤奋工作上。

　　各位代表！回顾过去，我们创造了令人难忘的业绩；展望未来，我们对鹤壁的前景充满信心！机遇难得需要奋力抢抓，使命光荣需要不懈拼搏，责任重大需要齐心共担。我们一定要保持清醒的头脑、振奋的精神、务实的作风，乘势而上，克难攻坚，锐意进取，不断开创经济社会发展新局面，全面加快富民强市步伐，努力建设更加幸福美好的明天，不辜负全市人民的期望和重托！

　　各位代表！人民的期待鞭策我们敬业尽责，宏伟的蓝图鼓舞我们奋勇前进。让我们在省委、省政府和市委的正确领导下，进一步解放思想、务实创新，同心同德、扎实工作，全面完成今年的各项目标任务，实现“十二五”良好开局，为创建科学发展示范区、实现率先崛起而努力奋斗！
